# Impacts of the advancement in artificial intelligence on laboratory medicine in low‐ and middle‐income countries: Challenges and recommendations—A literature review

**DOI:** 10.1002/hsr2.1794

**Published:** 2024-01-04

**Authors:** Malik Olatunde Oduoye, Eeshal Fatima, Muhammad Ali Muzammil, Tirth Dave, Hamza Irfan, F. N. U. Fariha, Andrew Marbell, Samuel Chinonso Ubechu, Godfred Yawson Scott, Emmanuel Ebuka Elebesunu

**Affiliations:** ^1^ Medical Research Circle Bukavu Democratic Republic of Congo; ^2^ Services Institute of Medical Sciences Lahore Pakistan; ^3^ Dow University of Health Sciences Karachi Pakistan; ^4^ Bukovinian State Medical University Chernivtsi Ukraine; ^5^ Shaikh Khalifa Bin Zayed Al Nahyan Medical and Dental College Lahore Pakistan; ^6^ Médecins Sans Frontieres Maiduguri Nigeria; ^7^ School of Public Health Yale University New Haven Connecticut USA; ^8^ Department of Medical Diagnostics Kwame Nkrumah University of Science and Technology Kumasi Ghana; ^9^ University of Nigeria Enugu Nigeria

**Keywords:** artificial intelligence, laboratory medicine, low‐ and middle‐income countries

## Abstract

**Background and Aims:**

Artificial intelligence (AI) has emerged as a transformative force in laboratory medicine, promising significant advancements in healthcare delivery. This study explores the potential impact of AI on diagnostics and patient management within the context of laboratory medicine, with a particular focus on low‐ and middle‐income countries (LMICs).

**Methods:**

In writing this article, we conducted a thorough search of databases such as PubMed, ResearchGate, Web of Science, Scopus, and Google Scholar within 20 years. The study examines AI's capabilities, including learning, reasoning, and decision‐making, mirroring human cognitive processes. It highlights AI's adeptness at processing vast data sets, identifying patterns, and expediting the extraction of actionable insights, particularly in medical imaging interpretation and laboratory test data analysis. The research emphasizes the potential benefits of AI in early disease detection, therapeutic interventions, and personalized treatment strategies.

**Results:**

In the realm of laboratory medicine, AI demonstrates remarkable precision in interpreting medical images such as radiography, computed tomography, and magnetic resonance imaging. Its predictive analytical capabilities extend to forecasting patient trajectories and informing personalized treatment strategies using comprehensive data sets comprising clinical outcomes, patient records, and laboratory results. The study underscores the significance of AI in addressing healthcare challenges, especially in resource‐constrained LMICs.

**Conclusion:**

While acknowledging the profound impact of AI on laboratory medicine in LMICs, the study recognizes challenges such as inadequate data availability, digital infrastructure deficiencies, and ethical considerations. Successful implementation necessitates substantial investments in digital infrastructure, the establishment of data‐sharing networks, and the formulation of regulatory frameworks. The study concludes that collaborative efforts among stakeholders, including international organizations, governments, and nongovernmental entities, are crucial for overcoming obstacles and responsibly integrating AI into laboratory medicine in LMICs. A comprehensive, coordinated approach is essential for realizing AI's transformative potential and advancing health care in LMICs.

## 1 INTRODUCTION

In laboratory medicine, artificial intelligence (AI) has become a revolutionary technology with the potential to completely change many facets of healthcare delivery. AI refers to the ability of a computer system to learn, think, and make decisions, tasks that typically require human intelligence. This technology enables computers to mimic human cognitive functions, such as understanding information, reasoning through complex situations, and arriving at conclusions. AI is being used in laboratory medicine to improve accuracy and efficiency, streamline and improve diagnostic processes, and help in clinical decision‐making.^1^, ^2^ The importance of AI developments in laboratory medicine resides in its capacity to analyze enormous amounts of data, spot trends, and produce useful insights swiftly and accurately. AI can help in interpreting laboratory test findings, forecasting patient outcomes, and assisting in the early diagnosis of diseases by utilizing machine learning (ML) algorithms.^1^ This technology has the potential to increase patient outcomes, decrease errors in diagnosis, and optimize treatment plans.^1^, ^2^

AI is making a significant impact in the field of medical imaging, revolutionizing diagnostics, and patient care. AI algorithms have the capability to achieve a high level of precision in detecting pathologies present in radiological images like X‐rays, computed tomography scans, and magnetic resonance imaging scans.^3^ As a result, diseases like cancer can be detected earlier, allowing for quicker and more efficient treatment. To improve the precision and effectiveness of diagnosis, pathologists can use AI to analyze histological images.^2^ Predictive analytics is a prominent use of AI in laboratory medicine. AI algorithms are able to pinpoint risk factors, forecast illness progression, and direct decisions on individualized treatment by analyzing sizable data sets containing patient data, laboratory results, and clinical outcomes.^1^ This could enhance patient care, improve treatment plans, and support the development of precision medicine techniques.^4^

AI technology has the potential to assist healthcare systems in low‐ and middle‐income countries (LMICs). The term “low‐ and middle‐income countries” is used to group nations according to their level of development and economic standing. Based on their Gross National Income per capita, the World Bank divides nations into a range of income groups, including low‐income, lower‐middle‐income, and upper‐middle‐income countries.^1^, ^5^ Numerous socioeconomic factors, such as income levels, poverty rates, the state of the healthcare system, and accessibility to resources and services, define LMICs. For AI to improve in laboratory medicine, LMICs are essential. These nations frequently experience difficulties regarding health care, such as few resources, poor infrastructure, and a high burden of diseases.^4^, ^6^ AI technologies can solve these problems and enhance the delivery of health care in LMICs. For instance, AI can support clinical decision‐making in resource‐constrained environments, improve the accuracy of illness identification, and optimize diagnostic processes.^1^, ^3^ LMICs can address disparities in health care, improve patient outcomes, and overcome limitations in access by making use of AI algorithms.^1^, ^6^ The need for accessible and inexpensive healthcare solutions is intimately related to the development of AI in laboratory medicine in LMICs.^4^, ^6^

In addition to enhancing disease surveillance and enabling earlier disease detection and management, AI algorithms can also help with the interpretation of laboratory test data as well.^1^, ^4^ It is significant to note that overcoming numerous obstacles is necessary for the successful integration of AI in laboratory medicine in LMICs. These difficulties include the scarcity of data, the requirement for contextually and locally appropriate algorithms, moral issues, capacity building, and infrastructure development.^1^, ^6^ Cooperation and collaboration are essential for knowledge sharing, resource sharing, and capacity development in the area of AI in laboratory medicine between LMICs and high‐income nations, international organizations, and research institutes.^4^, ^6^

With a focus on LMICs, this review aims to explore the background, significance, and implications of AI advancements in laboratory medicine. An in‐depth analysis will be conducted to explore the background and significance of AI advancements in laboratory medicine, with a particular focus on their implications for LMICs. This review will provide potential solutions to overcome challenges and enhance healthcare outcomes in settings with limited resources by the use of AI. By assessing the current state of laboratory medicine in LMICs and identifying prevalent issues, valuable insights will be offered to policymakers, healthcare professionals, and researchers, contributing to informed decision‐making and the advancement of knowledge in the field.

## 2 OVERVIEW OF LABORATORY MEDICINE IN LMICS

In terms of illness identification, surveillance, control, and management, laboratory medicine is essential. However, LMICs still face major barriers to accessing quality‐assured laboratory diagnostics, which can lead to delayed or incorrect diagnoses and inefficient treatments.^7^, ^8^ These difficulties result of a number of reasons, including poor supply chain management, a lack of government standards for laboratory testing, inadequate infrastructure, and scarce laboratory supplies and equipment.^7^, ^8^ There have been initiatives to address these issues and advance laboratory medicine in LMICs in recent years. The Lancet Series on Pathology and Laboratory Medicine (PALM) in LMICs provides comprehensive investigations into the challenges and deficiencies within PALM services while also proposing strategies for enhancing laboratory medicine.^7^, ^8^ The recommended solutions involve improving infrastructure, ensuring necessary laboratory supplies and equipment are accessible, strengthening the workforce through mentoring and training efforts, establishing quality management systems, and setting government standards for laboratory testing.^9^ Additionally, programs like the Lancet Commission on Diagnostics stress the necessity of enhancing LMICs' access to diagnostics, particularly laboratory testing. The commission supports technology improvements and evidence‐based essential diagnostics lists, with a focus on COVID‐19, antimicrobial resistance, and global health security.^10^ Enhancing mentorship programs, making investments in infrastructure development, expanding possibilities for education and training, and putting in place efficient quality assurance mechanisms are all necessary to strengthen laboratory medicine in LMICs.^7^, ^11^ For information exchange, resource sharing, and capacity building, cooperation between LMICs, high‐income nations, international organizations, and research institutions is crucial.^7^

### 2.1 Present situation and issues in laboratory medicine

For LMICs, the development of AI in laboratory medicine brings both potential and challenges. Due to poor infrastructure, a lack of digitalization, and a disorganized healthcare system, access to comprehensive and high‐quality data may be restricted in LMICs.^12^ There is also a shortage of knowledge, resources, and specialists to implement AI.^13^ Maintaining patient trust and protecting sensitive data require ensuring data privacy, security, and confidentiality, which can pose challenges related to ethical and legal considerations.^14^

Despite these difficulties, attempts are being made to remove the obstacles to the implementation of AI in LMICs' laboratory medicine. Harnessing the potential of AI requires investing in digital infrastructure, establishing data‐sharing networks, and standardizing data.^15^ Additionally, creative finance structures and public–private collaborations may make AI technology easily available and inexpensive in LMICs.^5^

### 2.2 Significance of affordable and accessible diagnostic technologies

In LMICs, accessible and affordable diagnostic technologies are essential for the development of AI in laboratory medicine. A summary of the importance of these diagnostic techniques is provided in Table 1. There are various reasons why these technologies are crucial.

**Table 1:** Importance of affordable and accessible diagnostic technologies.

Significance	Reasons
1. Prompt disease detection and monitoring	Early and accurate disease detection to manage health care effectively in LMICs^9^
	Prevention of disease progression, reduced complications, and improved patient outcomes^16^
2. Targeted and customized treatments	Enables effective resource utilization and maximizes scarce healthcare resources^16^
	Informed decisions by healthcare professionals lead to cost savings and less wasteful interventions^16^
3. Integration with AI and machine learning	Provides precise and thorough data inputs for training and validation of AI algorithms^16^
	Enables the development of AI‐driven diagnostic tools for improved accuracy, speed, and efficiency^17^
4. Investment in research and development	Prioritizing affordable solutions tailored to LMICs' unique requirements^18^
	Encourages the creation and dissemination of accessible diagnostic tools^18^
5. Collaborative approaches for accessibility	Collaborations among governmental entities, healthcare institutions, and technology suppliers^18^
	Enhances affordability, accessibility, and sustainability of diagnostic technologies^18^

Abbreviations: AI, artificial intelligence; LMICs, low‐ and middle‐income countries.

First, the accessible and affordable diagnostic tools make it possible to promptly and accurately detect and monitor diseases, which are essential for managing health care in LMICs.^9^ These advancements simplify the diagnosis of diseases at an early stage, thereby assisting in halting disease progression, reducing complications, and improving patient outcomes. Moreover, by enabling individualized and customized treatments, these breakthroughs promote the efficient use of resources. Optimizing the utilization of limited healthcare resources is crucial. Accessible and affordable diagnostic tools in LMICs empower healthcare practitioners to make informed choices, leading to efficient treatments, reduced unnecessary interventions, and cost‐effective measures.^16^

Additionally, these technologies enable the incorporation of ML models and AI algorithms into laboratory medicine operations. AI requires accurate and detailed data for training and validation, which can be generated by accessible diagnostic technologies.^16^ This combination allows the creation of AI‐driven diagnostic tools that enhance accuracy, speed, and efficiency in diagnoses,^17^ such as automated image analysis systems and predictive models. Making accessible and affordable diagnostic tools a priority is crucial for fully harnessing the potential of AI in laboratory medicine within LMICs. This requires allocating funds for research and development aimed at generating cost‐effective solutions tailored to the distinctive needs of LMICs. The deployment of these technologies can also be made more affordable, accessible, and sustainable through collaborations between governmental entities, healthcare institutions, and technology suppliers.^18^

### 2.3 The role of laboratory Medicine in healthcare delivery

In LMIC nations, laboratory medicine is crucial to the provision of health care. It supports healthcare systems in several ways, including:

First and foremost, laboratory medicine offers crucial diagnostic services that support the early detection, diagnosis, and follow‐up of diseases. For directing clinical decision‐making, treatment selection, and patient management, accurate and rapid laboratory testing is essential.^8^ These examinations cover a wide range of topics, such as cellular pathology, microbiology, clinical chemistry, and hematology. The outcomes of these tests provide medical practitioners with information about a patient's health status and direct the best course of action.

Additionally, laboratory medicine plays a vital role in monitoring and managing illnesses. The data it provides about disease prevalence, outbreaks, and patterns enables public health authorities to effectively track and address health risks. Through the identification and monitoring of infectious diseases, laboratory medicine also supports the implementation of preventive measures such as vaccination campaigns and strategies for infection control.^19^

Moreover, laboratory medicine serves as the basis for research studies, clinical trials, and the creation of innovative diagnostic tools and technologies. Research carried out in LMICs can solve regional healthcare issues and increase knowledge globally in laboratory medicine.^8^ The role of laboratory medicine in delivering health care in LMICs is very important for reducing disease impact, ensuring accurate diagnosis, and improving patient outcomes.

## 3 ADVANCEMENTS IN AI IN LABORATORY MEDICINE

Only a few total laboratory automation (TLA) systems have been built, even though all contemporary clinical chemistry and hematology analyzers are highly automated. A key automated laboratory instrument from Roche Diagnostics, the Cobas®, can process, analyze, and store samples on its own. This device can also carry out sample sorting, decapping, quality control, aliquoting, and recapping with in vitro diagnostic specimen tubes when used in conjunction with one or more connection modules on a track system. The Accelerator, represents an extensively automated system designed for preanalytical operations that may be connected to a multiinstrument core laboratory system, and it was just introduced by Abbott Diagnostics. The Accelerator has the benefit of being “open” to enable integration with outside systems, facilitating the consolidation of testing across laboratory specialties. The Power Express Clinical Automation system (Beckman), TCAutomationTM (Thermo Fisher), Aptio® Automation (Siemens Healthineers), and VITROS® Automation Solutions (Ortho Biomedical) are more examples of commercially available TLA systems. These centralized methods have been shown to improve analyzer performance and decrease human error.^20^, ^21^, ^22^, ^23^, ^24^

The introduction of TLA to microbiology and the ensuing revolution in this field were made possible by recent developments in the digitalization of culture plate pictures and the capacity to electronically scan incubated plates.^20^ Clinical laboratories now use two commercially available microbiological automation systems: WASPLab® (Copan Diagnostics Inc.) and KiestraTM (Beckton Diagnostics). Different levels of automation are possible with both devices, ranging from front‐end processing alone (automatic plating) through TLA.^20^ Historically, the clinical laboratory's microbiology area has required a lot of physical labor. To increase productivity and lessen the continuous national scarcity of medical laboratory scientists, automated workflows have gained more attention in recent years.^25^ Opportunities exist for the incorporation of ML‐based tools in these new processes as a result of this change in practice.

Increased automation is advantageous because it reduces costs, frees up technicians to perform more skilled tasks (such as microscopy, plate interpretation, and antimicrobial susceptibility testing), improves efficiency (as measured by LEAN or other benchmarks), decreases turnaround time, improves performance, and consolidates a number of manual and laborious clinical microbiology processes.^20^, ^24^, ^26^

The field of molecular diagnostics has undergone a radical change thanks to the introduction of high‐throughput and high‐multiplexity nucleic acid technology. These techniques have been made possible in part by ML advancements. For instance, many next‐generation sequencing (NGS) techniques evaluate millions of small clusters of tagged nucleic acids in thousands of photos at their core. These techniques produce enormous amounts of data, which calls for robust big data management pipelines because people cannot analyze such large amounts of data on their own.^27^, ^28^, ^29^

High‐dimensional, organized data sets produced by modern NGS assays can offer incredibly helpful diagnostic and prognostic insights. The scale and complexity of these data sets, however, make the analysis of NGS data sets labor and time‐intensive. As a result, numerous software solutions employ ML to accelerate different steps in the NGS data analysis pipeline. This technology, like other ML applications, can be used to improve human interpretations or open up new diagnostic possibilities. These tools are helpful for variant calls, curation, and clinical interpretations.^30^

## 4 THE IMPACT OF AI ADVANCEMENT IN LABORATORY MEDICINE IN LMICS

There is a prevailing belief that the outcomes of laboratory tests significantly influence approximately 70% of decisions related to diagnosis, treatment, and patient discharge.^31^ Increasing workload, rising healthcare costs, and the need for greater precision necessitate the continuous enhancement of laboratory operations.^32^ The ability to provide precise, conveniently available, and contextualized data has become increasingly important in the health care and laboratory medicine sectors with the advent of big data and AI.^33^ The utilization of AI in laboratory medicine has the potential to bring about significant advancements, particularly for individuals residing in LMICs. An overview of the impacts of these advancements is presented in Table 2. These advancements include the enhancement of laboratory processes, utilization, and interpretation, as well as the improvement of precision and speed in the delivery of patient‐specific therapy. The field of AI in health care revolves around the examination of extensive quantities of medical data generated through diagnostics, medical records, claims, clinical trials, and similar sources. This examination is conducted through the utilization of advanced algorithms and software designed to replicate human cognitive processes.^1^ In order for AI algorithms to function optimally, it is imperative that the laboratory data utilized is both accurate and reliable.^37^

**Table 2:** Impacts of AI advancements in laboratory medicine in LMICs.

AI advancements	Impacts
Timely disease identification	AI analyses diverse data sets to timely identify diseases like sepsis, cancer, Alzheimer's^34^, ^35^
Disease prediction	AI uses mathematical models and machine learning to predict infectious disease occurrences and impacts^36^
Role of AI and big data	AI and big data provide precise, available, and contextualized data for health care^33^
Disease monitoring and identification	AI uses big data analytics and natural language processing to monitor disease patterns and identifies diseases promptly^34^
Health care and diagnostics	AI examines extensive medical data using advanced algorithms to replicate human cognitive processes^1^

Abbreviations: AI, artificial intelligence; LMICs, low‐ and middle‐income countries.

AI has emerged as a highly promising tool within the field of laboratory medicine, offering a wide array of current and potential applications. One notable application pertains to the forecasting of laboratory test values using clinical and demographic data, thereby enabling enhanced efficiency and individualized patient care.^38^ Furthermore, AI has demonstrated significant potential in enhancing the efficiency of laboratory utilization through the streamlining of test ordering procedures and the reduction of unnecessary testing.^38^ As a result, this technology has the ability to conserve resources and minimize healthcare expenditures. The field of laboratory processes is currently undergoing a significant transformation facilitated by the integration of AI. This integration has led to the automation of various tasks^39^ within laboratories, including sample preparation, analysis, and reporting. This not only improves precision and efficiency but also enables laboratory personnel to allocate their attention toward more intricate responsibilities. In recent times, there has been a growing inclination toward the utilization of automated workflows as a means to enhance efficiency and address the persistent shortage of medical laboratory scientists at a national level.^25^ AI has the potential to enhance the efficient allocation of scarce resources, including human resources, equipment, and supplies. This can be achieved through the provision of decision support, quality control, and predictive maintenance.^38^

An additional promising area for the application of AI is in the field of precise laboratory test interpretation. This involves the integration of genomic, proteomic, and metabolomic data to offer more extensive and personalized diagnostic insights.^38^ Moreover, the influence of AI extends to the enhancement of laboratory medicine information systems. This is achieved through the augmentation of data management, security, and interoperability, thereby ensuring the smooth integration and utilization of information. Within the discipline of pathology, algorithms powered by AI have demonstrated significant promise in the classification of central nervous system tumors by utilizing DNA methylation profiling.^40^ This holds the potential to improve diagnostic accuracy and optimize treatment approaches. Finally, the field of digital pathology has the potential to have substantial advantages through the utilization of AI for image analysis.^41^ This integration can provide valuable assistance in the areas of diagnosis, prognosis, and selection of treatment options.^40^

The utilization of big data analytics and natural language processing enables AI to assist in monitoring disease patterns and occurrences, facilitating a more accurate and prompt identification of diseases. AI has the capability to analyze vast and diverse data sets, including electronic health records, genomic data, imaging data, and social media data, to identify patterns and biomarkers that may indicate the presence or risk of disease.^34^ Through the examination of physiological signals or brain scans, AI has the potential to assist in the timely identification of various diseases, such as sepsis, cancer, and Alzheimer's.^34^, ^35^ AI has the capability to gather and analyze data from diverse sources such as news headlines, online forums, official reports, and scientific publications. This enables AI to effectively monitor disease patterns and outbreaks.^35^ The utilization of mathematical models and ML algorithms enables AI to assist in predicting the occurrence and impact of infectious diseases such as COVID‐19.^36^

Expert systems, chatbots, and mobile applications have the capability to engage in communication with patients and healthcare providers, providing them with guidance and decision support pertaining to diagnosis and treatment. This guidance is derived from evidence‐based guidelines or patient‐specific data. AI has the potential to assist in the diagnosis of skin lesions, eye diseases, and tuberculosis through the utilization of image recognition and deep learning algorithms.^34^ Robotics and AI have the potential to fundamentally change the medical sector. These changes have the potential to improve patient care, resource allocation, and public health outcomes within healthcare systems, such as the field of laboratory medicine in these regions, which are impeded by various challenges.

## 5 SUCCESSFUL AI APPLICATIONS IN LMICs

In recent times, the domain of AI has witnessed significant progress, enabling its successful incorporation across various sectors in LMICs. A summary of examples of successful implementation of AI techniques in laboratory medicine is provided in Table 3. In the context of research conducted in India, scholars employed AI techniques to examine chest X‐rays with the aim of achieving precise and prompt identification of tuberculosis, surpassing traditional diagnostic approaches. The AI system provided confidence scores for individual diagnoses, which proved to be beneficial for clinicians in terms of prioritizing cases and efficiently allocating resources.^42^ Similarly, a research investigation carried out in Thailand utilized AI to analyze digital images of Papanicolaou smears, effectively detecting anomalous cells that are suggestive of cervical cancer or precancerous lesions. The AI system exhibited superior levels of sensitivity and specificity when compared to human cytotechnologists, resulting in decreased workload demands and accelerated turnaround durations.^13^

**Table 3:** Examples of successful AI Implementations in LMICs.

AI implementation	Location	Application	Impact
TB detection using AI	India	Analyzing chest X‐rays for TB diagnosis	Precise and rapid identification, confidence scores aiding clinicians in resource allocation^42^
Cervical cancer detection using AI	Thailand	Analyzing Pap smear images for cancer detection	Higher sensitivity and specificity, reduced workload, and faster turnaround compared to human cytotechnologists^13^
Malaria parasite identification with AI	Kenya	Analyzing blood smear images for malaria detection	Precise identification, parasite density estimation for disease severity monitoring, and treatment evaluation^43^
Antibiotic resistance prediction	Brazil	Analyzing bacterial genomic data	Prompt and accurate prediction of antibiotic resistance/susceptibility^38^
Sickle cell disease identification using AI	Nigeria	Analyzing hemoglobin electrophoresis outcomes	Accurate disease identification, risk scores for clinical management, and counseling^1^

Abbreviations: AI, artificial intelligence; LMICs, low‐ and middle‐income countries; TB, tuberculosis.

Moreover, a research study carried out in Kenya showcased the utilization of AI in the analysis of blood smear images for the precise and swift identification of malaria parasites, surpassing the capabilities of traditional manual microscopy methods. Additionally, AI also provided estimates of parasite density, thereby demonstrating its potential for monitoring the severity of the disease and evaluating the effectiveness of treatment.^43^ Another research study conducted in Nigeria used AI to analyze hemoglobin electrophoresis outcomes, resulting in the accurate identification of sickle cell disease with a higher level of precision compared to conventional approaches. AI also supplied risk scores for each diagnosis, offering valuable insights for the purposes of clinical management and counseling.^1^ Similarly, a study carried out in Brazil used AI to examine genomic data derived from bacteria, which enabled the anticipation of antibiotic resistance or susceptibility. The AI system demonstrated superior performance in comparison to conventional approaches that rely on phenotypic testing or molecular markers, providing prompt and comprehensive results.^38^ These cases demonstrate the feasibility of AI implementation in LMICs and highlight its pivotal role in addressing significant socioeconomic challenges within these regions.

Addressing the multifaceted challenge of bridging the digital divide and ensuring equitable access to AI technologies in LMICs demands concerted efforts from various stakeholders. One pivotal strategy involves investing in data infrastructure, governance, and literacy while promoting data sharing and interoperability. Tailoring AI solutions to the specific needs and contexts of diverse regions and populations, with consideration for cultural, ethical, and social values, is also imperative.^44^ Another critical aspect involves narrowing the skills gap through lifelong learning opportunities, fostering digital and AI skills, and encouraging creativity and critical thinking. Ensuring inclusive education, especially for women and girls, is vital to overcome disparities in digital inclusion.^44^, ^45^ Addressing public awareness and trust issues involves creating participatory communication platforms, enhancing AI system transparency, accountability, and explainability, and safeguarding personal data privacy and security.^44^, ^45^ To tackle ethical and social implications, AI technologies must be designed to empower human capabilities and uphold human values and rights. Implementing clear and consistent ethical frameworks and guidelines based on international norms and best practices is essential to guide the development and deployment of AI technologies across LMICs while instilling ethical awareness and responsibility among all stakeholders.^44^, ^45^

Therefore, fostering ethical deployment and bridging the technological divide are critical for LMICs to fully harness the potential of AI. Collaborative efforts addressing data availability, skills development, public awareness, and ethical considerations will pave the way for equitable AI integration. Tailoring AI solutions to local needs and values while ensuring transparency will enable LMICs to leverage AI's transformative power for sustainable development and inclusive growth.

## 6 CHALLENGES IN IMPLEMENTING AI IN LABORATORY MEDICINE IN LMICS

The discovery of the best medicines and preventive care may be aided by AI models, which have the potential to increase precision and speed in personalized medicine.^2^ However, there are various hurdles to implementing AI in laboratory medicine in LMICs. These difficulties can be examined in depth by addressing infrastructure, resources, personnel, data management, and cultural concerns.

LMICs frequently experience infrastructure constraints when it comes to AI implementation in laboratory medicine. Inadequate laboratory facilities, unstable power supply, limited internet connectivity, and insufficient hardware and software resources are examples of this. There is a scarcity of skilled professionals who can use AI tools and technology for diagnostics and analysis successfully.^5^, ^7^ The lack of necessary infrastructure and supply impedes the implementation and longevity of AI technology in laboratory medicine.

Another hindrance is limited access to quality data for AI training. For training, AI models require huge amounts of high‐quality data. However, due to limited resources, fragmented health information systems, lack of interoperable electronic health records, and inadequate data management practices, LMICs frequently experience difficulties in acquiring complete and diverse data sets.^5^, ^7^, ^46^ Inadequate data for training AI systems can result in subpar performance and generalizability. Concerns about data privacy and security, as well as a lack of data governance policies, might exacerbate data management in LMICs.

Implementing AI in laboratory medicine also heavily depends on ethical issues and legal frameworks. Using AI in laboratory medicine poses ethical and legal concerns, such as patient privacy, data protection, informed consent, and liability concerns.^4^ Because LMICs may lack well‐established policies and frameworks to address these problems, uncertainty and possible hazards may arise. To address difficulties with bias, transparency, responsibility, and liability, strong ethical principles and legislative frameworks tailored to AI in health care are required.

There also comes contextual bias since AI is being developed and deployed extensively in high‐income countries, and because the data utilized to create AI systems is highly tied to the context of use, adopting such systems in LMICs may result in contextual bias.^4^ In LMICs, integrating AI technologies with existing laboratory information systems might be difficult. Legacy technologies, interoperability difficulties, and a lack of technical experience impede seamless data interchange and integration between AI systems and laboratory procedures.^4^

Limited access to quality‐assured laboratory diagnosis poses a hindrance. Access to high‐quality laboratory diagnosis is critical for disease identification, surveillance, and treatment. Many LMICs, however, encounter obstacles due to a lack of critical infrastructure, laboratory supplies, basic equipment, experienced employees, and quality management systems.^5^, ^7^ These restrictions have an impact on the dependability and accuracy of laboratory data, which can have an impact on patient care.

In LMICs, limited resources, poor staff, and capacity restrictions lead to an insufficient amount and quality of laboratory services.^7^ A scarcity of trained laboratory professionals, such as pathologists and technicians, may hinder AI system installation. The expense of deploying and sustaining AI systems might be a substantial problem for LMICs with limited financial resources. AI technologies frequently necessitate large investments in infrastructure, software, and ongoing updates. Sustainable funding models and cost‐effective solutions are required to ensure the long‐term feasibility of AI implementation in laboratory medicine.^5^ Pathology and laboratory medicine are frequently undervalued in LMICs, resulting in insufficient investment, restricted resources, and a lack of knowledge of their significance.^5^, ^7^, ^47^ This lack of funding and awareness impedes the development and application of AI technologies in laboratory medicine.

The lack of resources and knowledge for implementing AI is another barrier. Data scientists and AI specialists who can create, implement, and manage AI systems are frequently in short supply in LMICs.^13^ Additionally, for environments with limited resources, the expense of adopting AI technology and integrating it into current laboratory operations can be prohibitive.^13^ The difficulties of implementing AI are further exacerbated by funding restrictions and competing healthcare agendas.

Addressing these issues would necessitate a multifaceted strategy. It entails developing and strengthening laboratory infrastructure, improving data collection and management systems, establishing ethical and legal frameworks, promoting capacity‐building and training programs, and raising awareness of the importance of pathology and laboratory medicine among policymakers and stakeholders.^4^, ^5^, ^7^

## 7 ETHICAL CHALLENGES AND CONSIDERATIONS IN AI ADOPTION

The field of AI ethics is closely intertwined with the fundamental issue of how those involved in the development, production, and operation of AI systems should conduct themselves to mitigate the ethical challenges that AI may pose within human civilization. These challenges can arise from unethical design, inappropriate implementation, or misuse of AI technology.^48^

### 7.1 Data privacy and security concerns in LMICs

AI applications in laboratory medicine that necessitate access to vast amounts of sensitive data, including patient records, laboratory results, and genomic information.^40^ Data privacy and security are imperative to safeguard the rights and interests of patients, healthcare providers, and researchers.^40^ However, LMICs may encounter obstacles in this regard due to the lack of adequate infrastructure, regulations, and awareness. For instance, insufficient data protection laws, encryption standards, and consent mechanisms can leave data vulnerable to unauthorized access, misuse, or breaches. Moreover, concerns surrounding data privacy and security may impede data sharing and collaboration among various stakeholders, such as AI technology providers, laboratory professionals, clinicians, and researchers. Consequently, it is crucial for LMICs to establish and implement appropriate policies and practices to address data privacy and security issues concerning the adoption of AI in laboratory medicine.

### 7.2 Capacity building and training programs

To successfully adopt AI in laboratory medicine, comprehensive capacity‐building and training programs for laboratory professionals, clinicians, researchers, and policymakers are essential.^49^ These programs enhance stakeholders' knowledge, skills, and competencies in utilizing, evaluating, and regulating AI applications in laboratory medicine.^40^ However, LMICs may face challenges in providing such programs due to limited resources, expertise, and infrastructure. For instance, inadequate funding, personnel, and equipment may hinder the implementation of capacity‐building and training initiatives for AI in laboratory medicine. Additionally, language barriers, cultural differences, and geographical constraints can further complicate the efforts to establish effective training programs. In light of these challenges, LMICs must leverage existing resources and establish partnerships to design and deliver impactful capacity‐building and training programs that foster the successful integration of AI in laboratory medicine.^40^

## 8 RECOMMENDATIONS FOR EMBRACING AI IN LABORATORY MEDICINE IN LMICS

### 8.1 Strengthening the infrastructure and capacity building

LMICs should prioritize investments in enhancing their infrastructure and capacity building to effectively adopt AI in laboratory medicine.^50^ Infrastructure encompasses physical components such as hardware, software, networks, devices, and sensors, as well as organizational components such as policies, standards, protocols, governance structures, and quality assurance systems. Capacity‐building initiatives should focus on human resource development, including education, training, certification, accreditation, mentoring, and career advancement for laboratory professionals, clinicians, researchers, and policymakers engaged in AI applications in laboratory medicine. By bolstering infrastructure and capacity, LMICs can improve the quality, accessibility, affordability, and sustainability of laboratory services and outcomes.

### 8.2 Collaboration and partnership with AI technology providers

Facilitating collaboration and forming partnerships with AI technology providers is crucial for harnessing the potential of AI in laboratory medicine within LMICs. These AI technology providers include companies, institutions, or organizations that develop or offer AI solutions tailored for laboratory medicine, such as software platforms, algorithms, models, tools, or devices. Collaborative efforts and partnerships with AI technology providers enable LMICs to access, adapt, and implement AI solutions that are suitable, relevant, and affordable for their specific contexts. Such collaborations also foster data sharing, knowledge transfer, technical support, and innovation in AI applications in laboratory medicine. However, it is essential that these collaborations are built on mutual trust, respect, and benefit, guided by ethical principles and standards.

### 8.3 Ethical guidelines and regulations for AI adoption

LMICs should establish and implement context‐specific, evidence‐based, and stakeholder‐inclusive ethical guidelines and regulations for AI adoption in laboratory medicine. These guidelines and regulations serve to ensure that AI applications in laboratory medicine meet standards of safety, effectiveness, reliability, transparency, accountability, and respect for human dignity, rights, and values. Moreover, they should address potential risks and challenges associated with AI adoption, such as data privacy and security, bias and discrimination, liability and responsibility, social and cultural implications, and human–machine interaction.

### 8.4 Establishing data‐sharing networks and repositories

LMICs must create data‐sharing networks and repositories to support the successful adoption of AI in laboratory medicine. These data‐sharing networks and repositories are designed to collect, store, manage, analyze, and share substantial volumes of data from various sources, including laboratory tests, clinical records, genomic sequencing, imaging modalities, or wearable devices. Leveraging AI techniques like data mining, ML, deep learning, or natural language processing, these data‐sharing networks and repositories can generate valuable insights, discoveries, innovations, and solutions for laboratory medicine. However, it is vital to ensure data quality, interoperability, standardization, and security while adhering to ethical and legal requirements.

### 8.5 The role of international organizations, governments, and nongovernmental organizations (NGOs) in supporting AI initiatives

International organizations, governments, and NGOs play a pivotal role in supporting AI initiatives in laboratory medicine in LMICs. Organizations such as the World Health Organization (WHO), United Nations International Children's Emergency Fund (UNICEF), and Global Alliance for Vaccines and Immunization (GAVI) offer guidance, funding, advocacy, coordination, and evaluation for AI initiatives in laboratory medicine. Governments must provide leadership, policy, regulation, infrastructure, and resources to advance AI initiatives in laboratory medicine within LMICs. NGOs contribute technical assistance, capacity building, innovation, and community engagement to drive progress in AI applications in laboratory medicine. However, successful outcomes depend on collaborative efforts and alignment of interests among international organizations, governments, and NGOs to maximize the impact and sustainability of AI initiatives in laboratory medicine within LMICs.

## 9 CONCLUSION

In summary, this review article discusses the progress of AI in LMICs. AI technologies are having a transformative impact on LMICs in various sectors, including health care and medicine. LMICs have effectively utilized AI to surmount limitations in resources and infrastructure, thus facilitating the advancement of sustainable development. However, there are still many challenges to the advancement of AI but AI has the potential to address disparities and facilitate transformative societal development in LMICs through the facilitation of interdisciplinary collaborations, exchange of knowledge, and targeted investments. This article explains the significance of developing AI ecosystems tailored to LMIC contexts to foster a more equitable and inclusive global technological environment.

## AUTHOR CONTRIBUTIONS

**Malik Olatunde Oduoye**: Conceptualization; project administration; supervision; validation; visualization; writing — review & editing. **Eeshal Fatima**: Validation; visualization; writing — original draft; writing — review & editing. **Muhammad Ali Muzammil**: Validation; visualization; writing — original draft. **Tirth Dave**: Validation; visualization; writing — original draft. **Hamza Irfan**: Validation; visualization; writing — review & editing. **F. N. U. Fariha**: Validation; visualization; writing — original draft. **Andrew Marbell**: Funding acquisition; validation; visualization. **Samuel Chinonso Ubechu**: Funding acquisition; validation; visualization. **Godfred Yawson Scott**: Conceptualization; funding acquisition; resources; validation; visualization; writing — original draft; writing — review & editing. **Emmanuel Ebuka Elebesunu**: Validation; visualization. All authors have read and approved the final version of the manuscript.

## CONFLICT OF INTEREST STATEMENT

The authors declare no conflict of interest.

## TRANSPARENCY STATEMENT

The lead author Godfred Yawson Scott affirms that this manuscript is an honest, accurate, and transparent account of the study being reported; that no important aspects of the study have been omitted; and that any discrepancies from the study as planned (and, if relevant, registered) have been explained.

## Data Availability

Data are available on request from the authors.
